# *In vivo* conversion of astrocytes into oligodendrocyte lineage cells with transcription factor Sox10; Promise for myelin repair in multiple sclerosis

**DOI:** 10.1371/journal.pone.0203785

**Published:** 2018-09-13

**Authors:** Akram Mokhtarzadeh Khanghahi, Leila Satarian, Wenbin Deng, Hossein Baharvand, Mohammad Javan

**Affiliations:** 1 Department of Brain Sciences and Cognition, Cell Science Research Center, Royan Institute for Stem Cell Biology and Technology, ACECR, Tehran, Iran; 2 Institute for Pediatric Regenerative Medicine, University of California, Davis, School of Medicine, Sacramento, California, United States of America; 3 Department of Developmental Biology, University of Science and Culture, Tehran, Iran; 4 Department of Physiology, Faculty of Medical Sciences, Tarbiat Modares University, Tehran, Iran; Instituto Cajal-CSIC, SPAIN

## Abstract

Recent studies demonstrate that astroglial cells can be directly converted into functional neurons or oligodendrocytes. Here, we report that a single transcription factor Sox10 could reprogram astrocytes into oligodendrocyte-like cells, *in vivo*. For transdifferentiation, Sox10-GFP expressing viral particles were injected into cuprizone-induced demyelinated mice brains after which we assessed for the presence of specific oligodendrocyte lineage cell markers by immunohistofluorescence (IHF). As control, another group of demyelinated mice received GFP expressing viral particles. After 3 weeks, the majority of transduced (GFP+) cells in animals which received control vector were astrocytes, while in animals which received Sox10-GFP vector, the main population of GFP+ cells were positive for oligodendrocyte lineage markers. We also extracted primary astrocytes from mouse pups and purified them. Primary astrocytes were transduced *in vitro* and then transplanted into demyelinated brains for later fate mapping. After three weeks, *in vitro* transduced and then transplanted astrocytes showed oligodendrocyte progenitor and mature oligodendrocyte markers. Further confirmation was done by transduction of astrocytes with lentiviral particles that expressed Sox10 and GFP and their culture in the oligodendrocyte progenitor medium. The induced cells expressed oligodendrocyte progenitor cells (iOPCs) markers. Our findings showed the feasibility of reprogramming of astrocytes into oligodendrocyte-like cells *in vivo*, by using a single transcription factor, Sox10. This finding suggested a master regulatory role for Sox10 which enabled astrocytes to change their fate to OPC-like cells and establish an oligodendroglial phenotype. We hope this approach lead to effective myelin repair in patients suffering from myelination deficit.

## Introduction

Oligodendrocytes possess a pivotal role in ensheathing axons by myelin. Their pathology contributes to various disorders including multiple sclerosis (MS), white matter stroke, traumatic CNS injury, and age-related myelin loss. Replacing the oligodendrocytes is a promising approach for therapeutic applications, in particular the generation of autologous sources of oligodendrocyte progenitor cells (OPCs) is of great interest.

*In vivo* conversion of somatic cells into desired cell types is considered a proper approach to generate progenitor repair cells for therapeutic purposes with emphasis on organs that have a low capacity to regenerate. *In vivo* lineage reprogramming approaches have been demonstrated in the brain[1, 2], spinal cord [3], heart [4], and pancreas [5].

Astrocytes are the most plentiful cellular components of glial scars which develop following neural cell loss from degenerative diseases and traumatic injuries by reactivation of astrocytes and their secretions [6]. Previous studies demonstrated that astrocytes could be directly converted into neurons or stem-like cells by the forced expression of transcription factors in vitro [7–11], which highlights the capability of fate change of these somatic glial cells. Recently, several attempts have been made to convert astrocytes within the brain parenchyma to neurons by Sox2 [3, 12, 13], NeuroD1 [1], Ascl1 [14], and MicroRNA 302/367 [15]. In this strategy, instead of surgical removal of the glial scar, reactive astrocytes are converted into progenitor cells that contribute to tissue repair. Developmentally, astrocytes and oligodendrocytes are produced from glial progenitors and may be considered as differentiated cells with similar epigenetic states [16]. In previous reports conversion of astrocytes to myelinating cells was done using MicroRNA 302/367 and transcription factor Oct4 that were not specific to oligodendrocytes [17, 18]. The same MicroRNA also produced neuroblasts which had the capability to differentiate into mature neuron-like cells in normal brains [15] and animal models of neurodegeneration and Alzheimer’s disease [19, 20]. Therefore, in the current study, we attempted to locate a single transcription factor specific to oligodendrocyte lineage cells that had the capability for in vivo conversion of astrocytes into OPCs.

Sox10 is a transcription factor related to the sex determining region Y (SRY)-boxes gene family expressed constantly throughout OPC development into mature oligodendrocytes[21]. Numerous evidences suggest that Sox10 is as a master regulator in the developmental process of oligodendrocytes and activation of myelination genes [22–24]. This transcription factor in combination with NKX6.2 and Olig2 [25]or Olig2 and ZFP536 [26] has the capability to reprogram rodent fibroblasts into induced OPCs (iOPCs). Continuous expression of Sox10 in satellite cells of dorsal root ganglion via a transgene approach has led to ectopic development of oligodendrocytes [27]. While NFIA is known as a key astrocyte fate determinant factor, it has been reported that Sox10 suppresses astrocytic differentiation by an antagonistic relationship with NFIA and regulating the oligodendrocyte and astrocyte fate during development [28] In fact, Sox10 positive astrocytes have been hardly detected in corpus callosum and brain cortices [29].This reports also provide another explanation for using Sox10 to convert astrocytes to OPCs.

To the best of our knowledge, this is the first report of using forced expression of a myelination specific transcription factor, Sox10 that has induced astrocytes to reprogram into oligodendrocyte lineage cells.

## Materials and methods

### Viral particle preparation

The desired construct containing a CDS for the Sox10 gene was generated by ligation between a sox10 CDs (extracted from Tet-O-FUW-Sox10) and a pSSFV-IRES-GFP plasmid, to acquire the pSSFV-Sox10-IRES-GFP. Tet-O-FUW-Sox10 was a gift from Marius Wernig (Addgene plasmid # 45843). The schematic diagram for vectors are presented in Panel A in S1 Fig.

Confluent human embryonic kidney cells (HEK 293T) were transfected with pSSFV-*Sox10*-IRES-*GFP* or pSSFV-IRES-*GFP* constructs and packaging plasmids (pCMV-vsvg and pCMV-gp) using Lipofectamine 3000 (Life Technologies). At 48 and 72 h post-transfection the cell culture media was collected, filtered, and centrifuged to concentrate the viral particles. The collected particles were re-suspended in 100 μL PBS for subsequent infections.

### Induction of demyelination in animals

Male C57BL/6 mice, 7–8 weeks old, (Royan Institute) were housed in a climate-controlled room with access to food and water ad libitum. All experiments were performed in accordance with the international guide for the care and use of laboratory animals. The experimental procedures wereevaluated and approved by the Committee for Ethics in Animal Research at Royan Institute. Attempts were made to minimize the number of animals used and their suffering. For demyelination induction, mice were fed with 0.2% (w/w) oxalic acid bis(cyclohexylidenehydrazide) (cuprizone; Sigma-Aldrich, C9012) mixed into their normal chow for 12 weeks. We conducted the Y-maze test and histological studies to confirm the presence of behavioral impairment due to demyelination and extensive demyelination in their brains.

Spontaneous alternations within the Y-maze was tested prior to induction of demyelination and after the 12-week period of cuprizone administration in their chow. The Y-maze arms were marked A, B, and C. The number of overlapping entrance sequences (e.g., ABC, BCA) was defined as the number of spontaneous alternations. We calculated the percent of alternations as follows [30].

Percent of alternations = (number of alternations)/ (total number of arm entries-2) ×100.

The number of total arm entries was calculated as the index of ambulatory activity in the Y-maze. We excluded any animals with scores of less than six entries.

### Animal interventions

We evaluated the possible conversion of transduced cells into myelinating cells. The schematic diagram for animal interventions are presented in Panel B in S1 Fig. Mice with cuprizone induced demyelination were anesthetized with ketamine (100 mg/kg, i.p., GE Healthcare, Germany) and xylazine (5 mg/kg, i.p., Bayer, Germany), then placed in a stereotaxic frame in a flat skull position. Lentiviral particles (2 μl of concentrated solution) were injected into each corpus callosum at the following coordinates from the atlas of Paxinos and Watson: anteroposterior (AP) = -1.06, mediolateral (ML) = 1 from the bregma, and dorsoventral (DV) = 1.5 mm from the skull. Animals received a normal diet for 3 or 8 weeks after the injection.

We assessed the conversion of astrocytes to oligodendrocyte lineage cells by transducing purified astrocytes (see below) with pSSFV-Sox10-IRES-GFP or pSSFV-IRES-GFP viruses. After 48 h, we transplanted 1×10^5^ cells into the corpora callosa of the mice with demyelinated brains. The animals received immunosuppression by daily injections of cyclosporine A (20 mg/kg; Novartis Pharmaceuticals, East Hanover, NJ, USA) from 2 days before surgery until 3 weeks after transplantation.

### Tissue sampling and immunohistofluorescence (IHF) studies

Animals were deeply anesthetized and perfused transcardially with saline, followed by 4% paraformaldehyde (Sigma-Aldrich, P6148) prepared in PBS. Then, the brains were removed, post-fixed overnight in 4% PFA, and cryoprotected for 48 h in 30% sucrose. Samples were embedded in optimal cutting temperature (OCT) compound and serial 10 μm-thick coronal sections were cut using a cryostat (Leica Microsystems, CM 1850). The cut sections were stored at -20°C and then processed for immunohistofluorescence (IHF) analysis.

Sections were washed three times in PBS, then incubated in blocking solution that consisted of 0.1% Triton X-100 (Sigma-Aldrich, T8532), 1% bovine serum albumin, and 5% normal serum in PBS for 1 h at room temperature, followed by an overnight incubation with primary antibodies against NG2, Olig2, glial fibrillary acidic protein (Gfap), PdgfRα, Plp, Mbp or NeuN at 4°C. Sections were washed three times with PBS and incubated at room temperature for 2 h with the secondary antibody. List of antibodies is provided in S1 Table. After treatment with 4′, 6-diamidino-2-phenylindole (DAPI; Sigma-Aldrich; D-8417), the sections were again washed three times with PBS. For myelin staining with FluoroMyelin, sections were incubated with FluoroMyelin Red (Invitrogen F34652, 1/300) solution dissolved in PBS for 20 minutes at room temperature. Sections were coverslipped and then Images were obtained on an Olympus IX51 fluorescence microscope with a DP72 digital camera and analySIS LS Starter software version 3.2.

### Astrocyte isolation and culture

Astrocytes were isolated based on the procedure by Schildge et al. [31] with some modifications. After removal of the meninges, grey matter tissue from the cerebral cortexes of P3-P5 C57BL/6J mice was dissected and mechanically dissociated. The suspension was centrifuged for 5 min at 1000 rpm, re-suspended, and plated in a medium that contained DMEM/F12 (Invitrogen), 3.5 mM glucose (Sigma), 10% fetal bovine serum (Gibco), and 1% penicillin/streptomycin (Gibco). After 24 h, the contaminating oligodendrocyte precursor cells were removed by vigorous manual agitation. One week later, cells were passaged using and plated in fresh medium. The CD44+ cell population was purified from a primary culture of astrocytes by incubation with anti-CD44 antibody (BD550538) followed by MACS selection (Miltenyi Biotech, Germany). We confirmed the fate of extracted cells and the purity of the sorted astrocytes by immunocytofluorescence (S2 and S3 Figs).

### Transduction of astrocytes in culture

Purified astrocytes were transduced with prepared viral particles and kept in astrocyte media for an additional 48 h. Afterwards, transduced cells were seeded on 0.1 mg/ml poly-l-ornithine (Sigma-Aldrich; P4707) and 10 μg/ml laminin (Sigma-Aldrich; L2020) for in vitro induction or transplanted into animal brains for in vivo transdifferentiation. In culture, the induction medium was permissive to oligodendrocyte fate acquisition and consisted of DMEM/F12 (Invitrogen) supplemented with N2 (Invitrogen), B27 without vitamin A (Invitrogen), 2 mM Glutamax (Invitrogen), 200 ng/ml SHH (Royan Biotech), 20 ng/ml bFGF (Royan Biotech), and 20 ng/ml PDGF-AA (Royan Biotech). Cultures were fixed after 21 days and stained against the OPC markers, Olig2 and NG2, and neuronal marker Tuj1; after which we determined the percent of transduced (GFP+) cells positive for NG2, Olig2 and Tuj1.

### Data analysis

When required, we performed cell counts in several randomly selected views of all samples from each brain section. At least 8 sections were counted per animal and each animal group included 3 mice. For statistical comparison of the means of different groups, one-way ANOVA followed by the Tukey post-test was used except for the Y-maze data which we compared with the Wilcoxon signed-rank test for matched pairs. The results were expressed as mean±SEM. A probability of p<0.05 was considered statistically significant.

## Results

**Fig 1:**
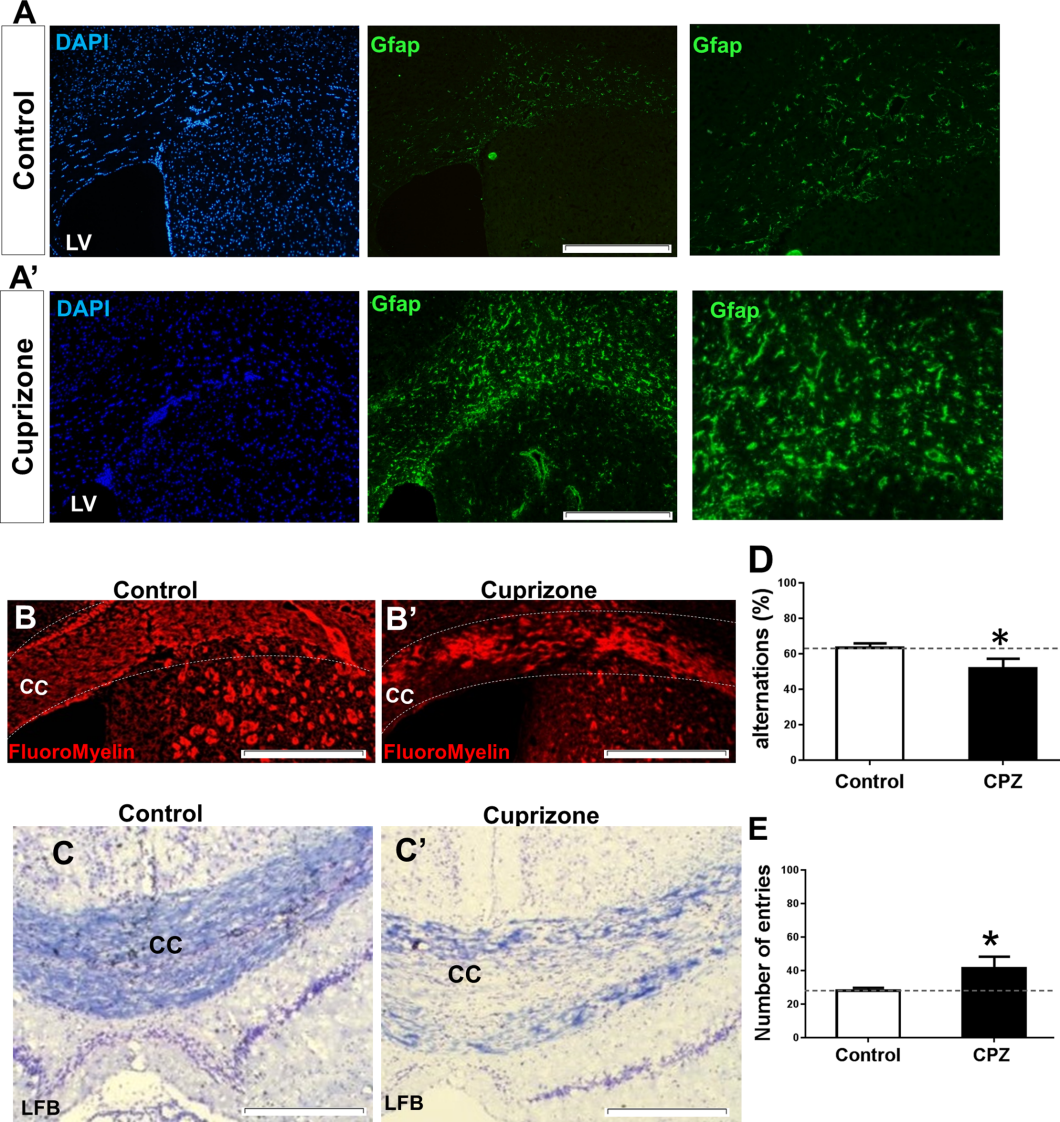
Confirmation of astrocyte reactivation and demyelination induction in mice brains treated with cuprizone. A) Immunohistofluorescence (IHF) staining for glial fibrillary acidic protein (Gfap) in control animals. Extensive astrogliosis was observed after 12 weeks of cuprizone feeding as mentioned in A. The nuclei were stained with DAPI. B) (FM) staining against myelin for the control (B) and cuprizone (B’) groups. C) Luxol fast blue (LFB) staining of myelin in control (C) and cuprizone treated animals (C’) showed extensive demyelination. D, E) Memory performance of control and cuprizone-treated mice. Spontaneous alternations (D) and number of total arm entries (E) was assessed using the Y-maze test. (n = 10). Horizontal lines show baseline amounts for each parameter. *: p<0.05 compared to the control groups (Wilcoxon matched-pairs signed rank test). LV: Lateral ventricle; CC: Corpus callosum. Scale bars: 200 μm.

Previous studies reported that a 12-week cuprizone diet led to extensive demyelination in the mice brains. This finding was particularly noted in the corpus callosum of each brain. Researchers have reported that a 12-week cuprizone diet decreased endogenous remyelination [32, 33] and therefore provides an adequate model to study the capability of *in situ* induced OPCs or transplanted cells for myelination. Astrogliosis, defined as astrocyte hypertrophy and reactivity, is one of the most important features of this demyelination model [34]. Gfap staining showed extensive astrocyte reactivation within the brains (Fig 1A). FluoroMyelin staining showed extensive corpus callosum demyelination in cuprizone-treated animals compared to control mice of the same age (20 weeks; Fig 1B). Demyelination was also confirmed by luxol fast blue staining (Fig 1C). Spatial working memory is affected following cuprizone induced demyelination in mice [35]. Measurements of spontaneous alternations within the symmetric Y maze confirmed the presence of myelination decline in the study mice. The treated mice showed significant impairment in terms of total arm entries and spontaneous alternations compared to baseline (Fig 1D and 1E).

**Fig 2:**
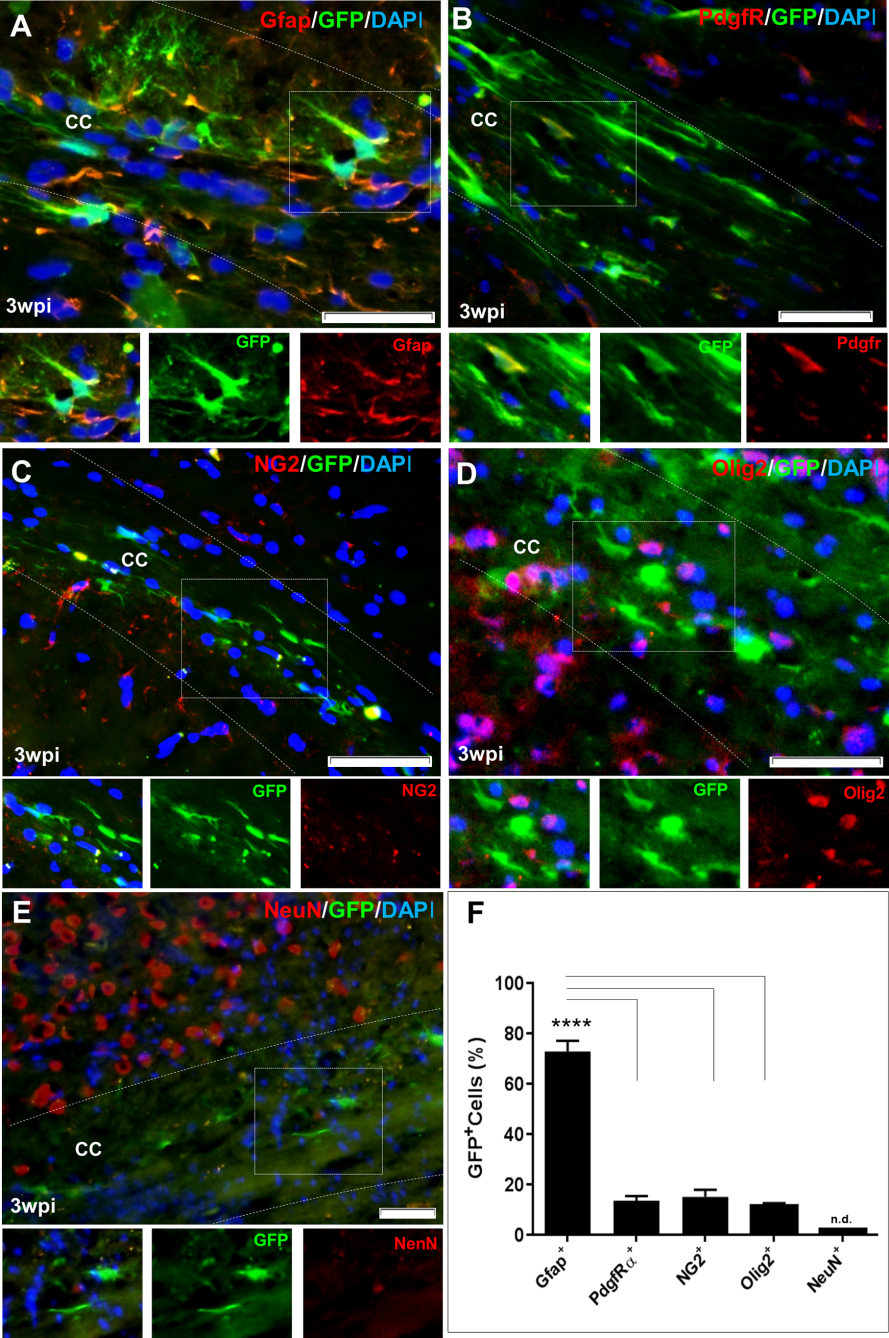
Determining the fate of cells transduced with pSSFV-IRES-GFP lentiviral particles injected into the corpus callosum. (A-E) Immunofluorescence staining against astrocytes marker Gfap, Oligodendrocyte lineage cell markers PdgfRα, NG2, Olig2 and neuron marker (NeuN) at 3 weeks post injection (wpi) of pSSFV-IRES-GFP lentiviral particles into the corpora callosa of demyelinated mice brains. Gfap+ astrocytes comprised the main population of transduced cells. (F) Quantified results for immunostaining against different markers used in A-E. Data are presented as mean±S.E.M. ***: p<0.001, n = 3 mice per group. Scale bar: 50 μm, n.d.: Non-detectable.

We designed a construct to express GFP and Sox10 under the control of the same promoter (pSSFV-*Sox10*-IRES-*GFP*) in order to detect the transduced cells (Panel A in S1 Fig) and injected as viral particles into the corpora callosa of the mice after confirmation of demyelination using Y maze. In order to determine the identity of transduced cells, the pSFFV-IRES-*GFP* lentiviral particles which label the transduced cells without changing their fate, were injected into the corpora callosa (Panel B in S1 Fig). After 21 days, the animals were sacrificed for brain sampling and IHF studies against different cell markers. Accordingly, most transduced cells tested positive for Gfap as a marker of astrocytes (Fig 2A), while oligodendrocyte lineage cells comprised a small number of transduced cells (Fig 2B–2D). There were no prominent co-expressions of NeuN (a neuronal marker) and GFP detected, which indicated that the neurons were not transduced. The lack of transduction appeared to be due to their distinct location from the injection site (Fig 2E). Following quantification, the proportion of different cell types within the transduced GFP+ cells were as follows: 72.75%±4.3 (Gfap+), 13.5%±1.8 (PdgfRα+), 15%±2.8 (NG2+), and 11.25%±1.3 (Olig2+). No NeuN+/ GFP+ cells were detected (Fig 2F). A part of above mentioned cells may be originated from the transduced progenitor cells which have been differentiated to astrocytes or oligodendroglial cells during 3 weeks chase time. Therefore the origin of transduced cells in this experiment remains not fully cleared.

**Fig 3:**
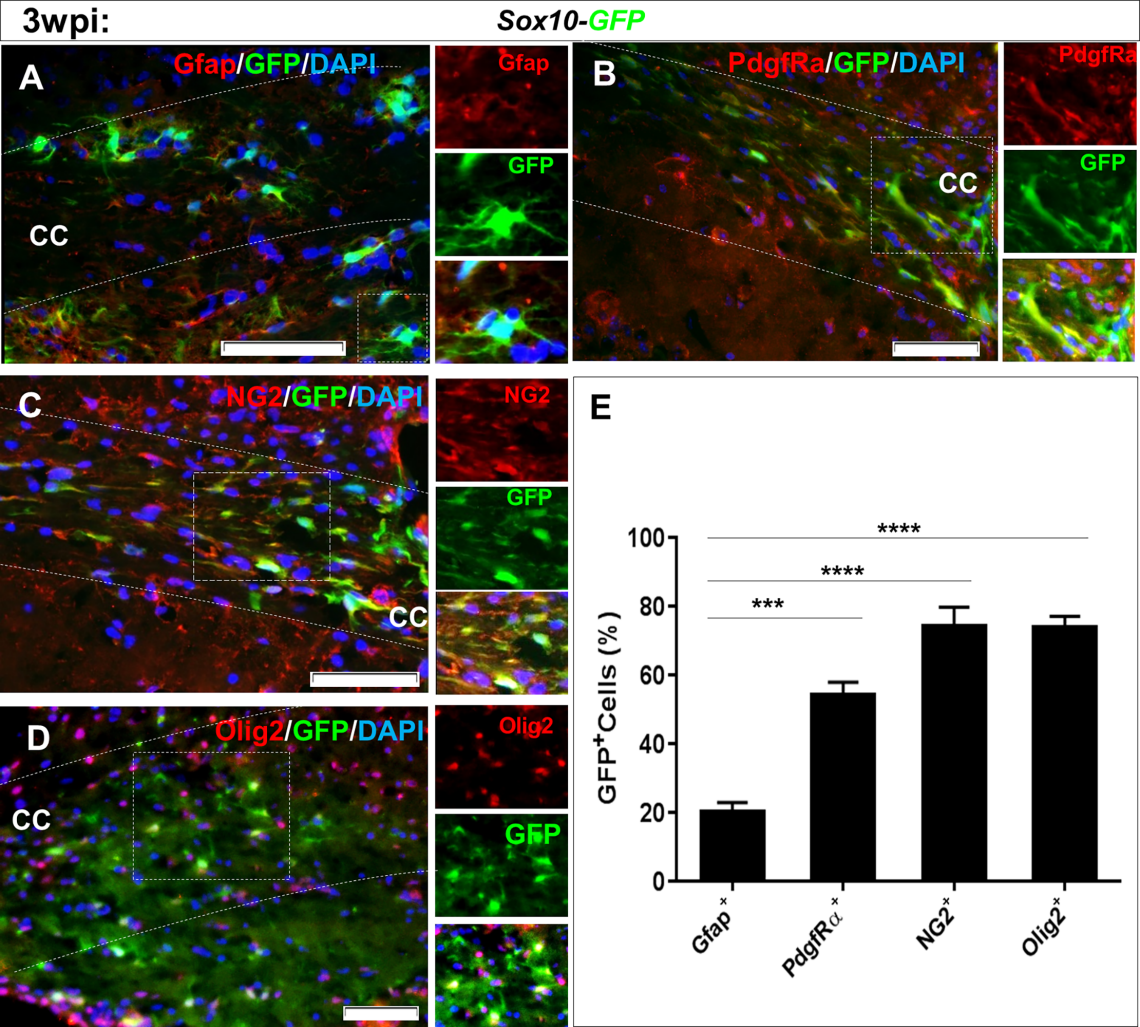
Changes in the fate of cells transduced with SSFV-Sox10-IRES-GFP lentiviral viruses in vivo. (A) Immunohistofluorescence staining showed lower number of GFP+ cell stained with Gfap. The expression of PdgfRα (B), NG2 (C), and Olig2 (D) markers in transduced cells within the corpus callosum, at 3 weeks post injection (wpi) was increased. GFP+ cells mainly expressed Olig2, PdgfRα, and NG2 (right panels show higher magnifications). E) Quantified results for immunostaining against different markers. Data are presented as mean±S.E.M. ***: p<0.001 compared to Gfap, n = 3 animals.

We investigated whether *Sox10* could convert astrocytes into oligodendrocyte lineage cells *in vivo*. Viral particles that contained the pSFFV-*Sox10*-IRES-*GFP* (*Sox10*-*GFP*) vector were directly delivered into the corpora callosa of the cuprizone induced demyelinated mice, where reactive astrocytes persisted. Mice were followed for 3 weeks after the injection. Ultimately, the brain samples were collected and prepared for sectioning and staining against astrocyte marker Gfap (Fig 3A), and oligodendrocyte markers PdgfRα (Fig 3B), NG2 (Fig 3C), and Olig2 (Fig 3D). Interestingly, as the representative micrographs showed, higher numbers of GFP+/Olig2+, GFP+/NG2+, and GFP+/PdgfRα+ cells compared to GFP+/Gfap+ cells were counted. Fig 3E shows the quantified data for the percentages of different GFP+ cell types. Within the GFP+ cells, we observed significantly more Olig2+, NG2+, and PdgfRα+ cells compared to Gfap+ cells (all: p<0.001). When compared the percentage of GFP+ cells with different labels to those mentioned in Fig 2 (animals which received only-GFP expressing vector), there was a significantly reduced number for Gfap+ cells (72.75% vs. 20%). The number of GFP+ cells stained with oligodendrocyte lineage cell markers were significantly increased by Sox10 expression, e.g. NG2+ cells increased from 15% to 74% which purpose possible conversion of astrocytes to oligodendrocyte lineage cells.

**Fig 4:**
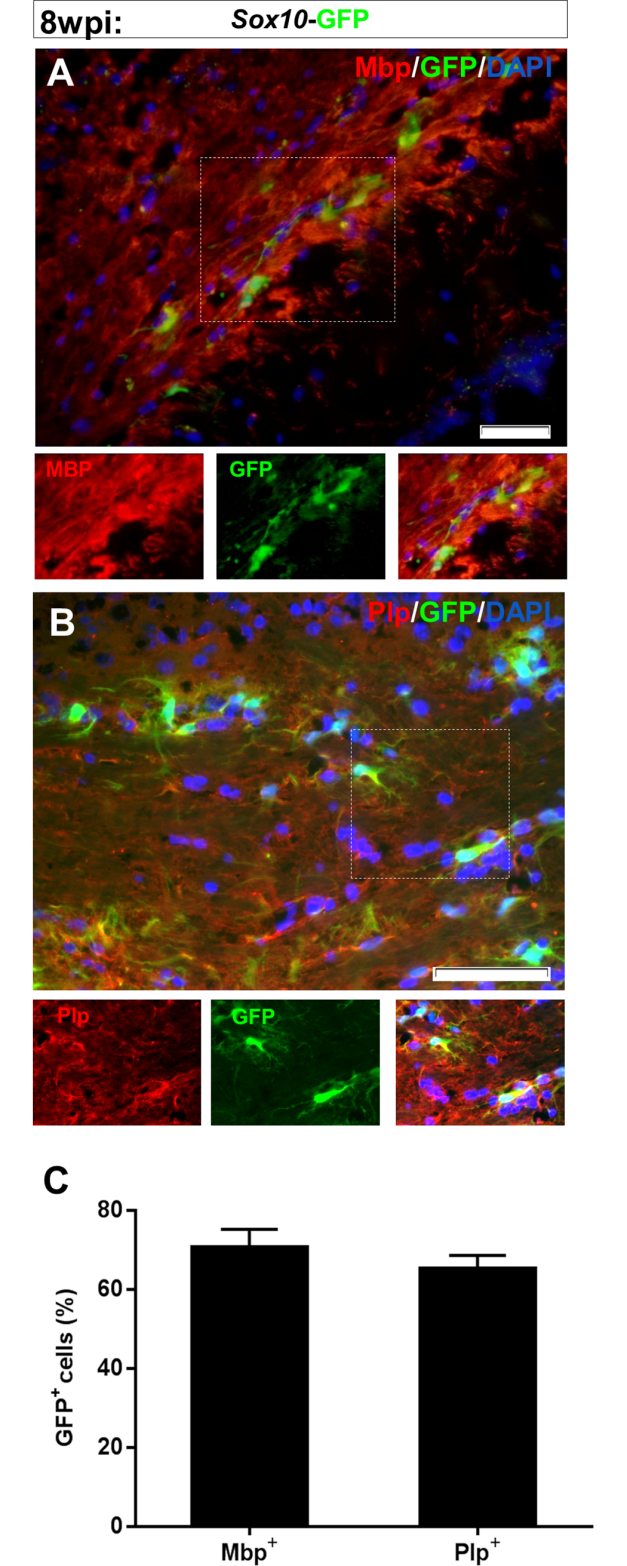
Expression of mature oligodendrocytes markers by in vivo transduced cells at 8 weeks post viral particles injection. GFP+ cells were mainly positive for Mbp (A) and Plp (B). C) Quantification of results for immunostaining against mature oligodendrocyte markers presented in A and B. Scale bars: 50 μm.

In another group of animals, *Sox10*-*GFP* expressing viral vectors were injected into the corpora callosa of demyelinated mice and the samples were collected 8 weeks after the injection. Staining against Mbp and Plp as markers of mature oligodendrocytes showed that transduced (GFP+) cells were mainly differentiated to oligodendrocytes-like cells (Fig 4A and 4B). Data quantification showed that 70.66±2.6 and 62.25± 3.3 percent of GFP+ cells were respectively Mbp+ and Plp+ (Fig 4C).

**Fig 5:**
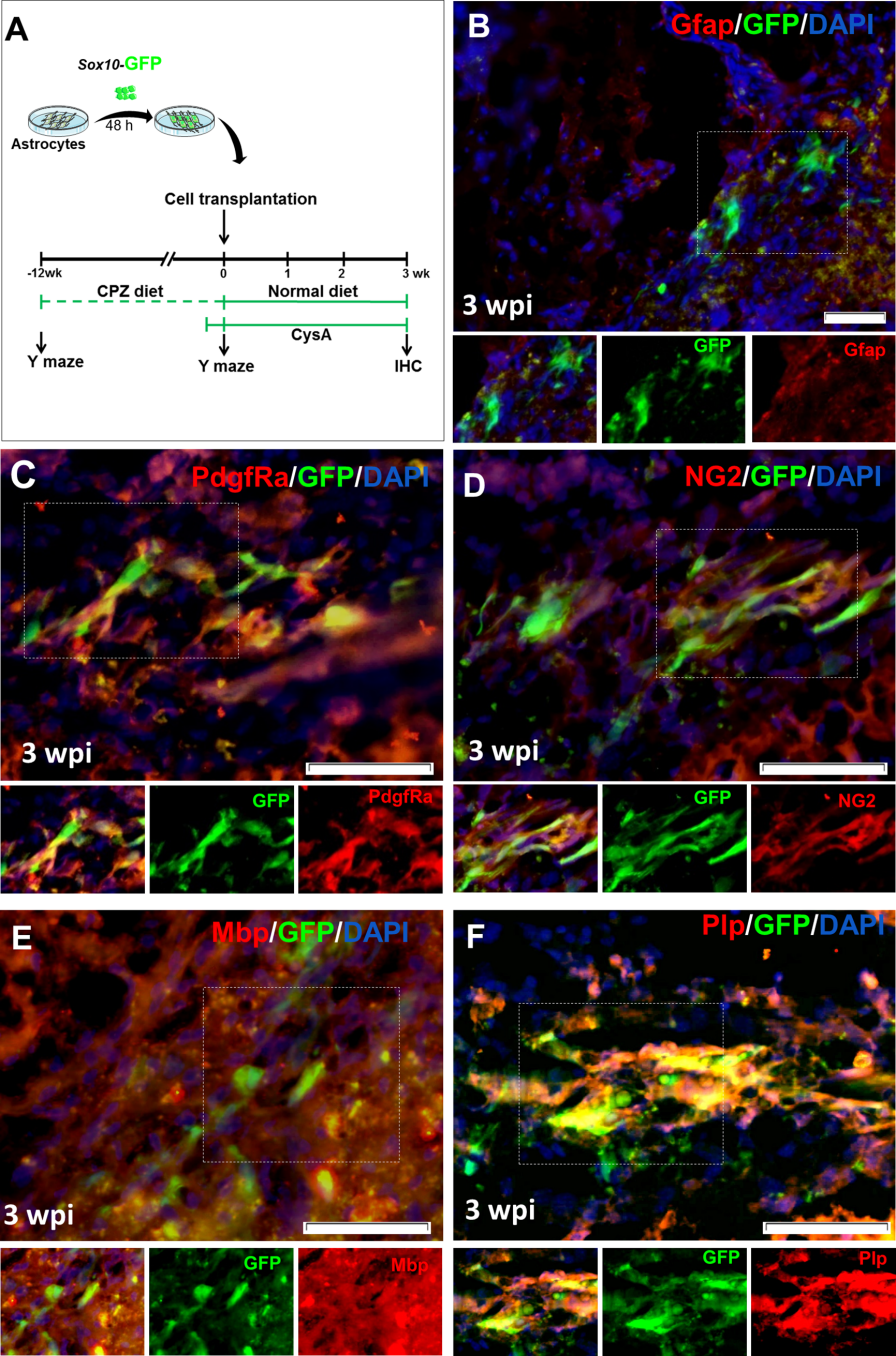
*In vivo* conversion of purified mouse astrocytes transduced with SSFV-Sox10-IRES-GFP lentiviral particles after transplantation into the corpora callosa of demyelinated mice brains. A) Schematic diagram of the experimental procedure of demyelination induction, Y maze test for data presented in Fig 1D and 1E, astrocyte transduction, and transplantation. B) Immunohistofluorescence (IHF) staining against Gfap showed the lack of notable co-expressions of GFP and Gfap as astrocyte marker at 3 weeks post injection (wpi). C-D) Expressions of PdgfRα (C) and NG2 (D) markers in the main population of GFP+ cells in the corpus callosum at 3 wpi. The bottom panels show higher magnifications. E-F) IHF showed that transplanted astrocytes (GFP+) could express mature oligodendrocyte markers Mbp (E) and Plp (F) at 3 wpi. n = 3 animals, CysA: cyclosporine A for preventing rejection of transplanted cells, Scale bars: 50 μm.

The previous experiment showed that local injection of Sox10-expressing vector into the demyelinated corpus callosum increased the number of oligodendrocytes lineage-like cells within the transduced cells. Since the origin of in vivo transduced cells was not fully clear, it was not possible to make a solid conclusion that astrocytes had the capability to differentiate to oligodendrocyte-like cells after receiving the Sox10 expression vector. Therefore, we planned to extract primary astrocytes, purify and transduce them with Sox10 expressing vector for subsequent transplantation into demyelinated mice corpora callosa. To prepare the starting cells, we isolated astrocytes from the brains of 3–5 day-old mice (S2 Fig). The primary culture cells underwent magnetic activated cell sorting against the CD44 cell surface marker to obtain a pure population of astrocytes. Astrocytes were negative for NG2, Sox10 and Olig2 as OPC and oligodendrocyte markers, and negative for Nestin as a marker for neural progenitors as evaluated by immunocytofluorescence (S3 Fig). We transduced astrocytes *in vitro* and subsequently transplanted them into the corpora callosa of the cuprizone-induced demyelinated brains (Fig 5A). After 3 weeks, the brain sections were stained for Gfap (astrocyte marker) and PdgfRα, NG2, Mbp, and Plp as markers of oligodendrocyte progenitors and mature oligodendrocytes. According to Fig 5B–5F, GFP+ cells expressed PdgfRα and NG2, as markers of OPCs, as well as Mbp and Plp, as markers of myelinating oligodendrocytes. A low number of GFP+ cells were still Gfap+. As we observed in 6 independent animals, following transplantation, GFP only-transduced astrocytes could not survive at 3 weeks after transplantation. These data may provide an evidence that Sox10 but not GFP (as control) caused in vivo trans-differentiation of astrocytes to oligodendrocyte lineage cells which were capable to integrate into the tissue and survive.

**Fig 6:**
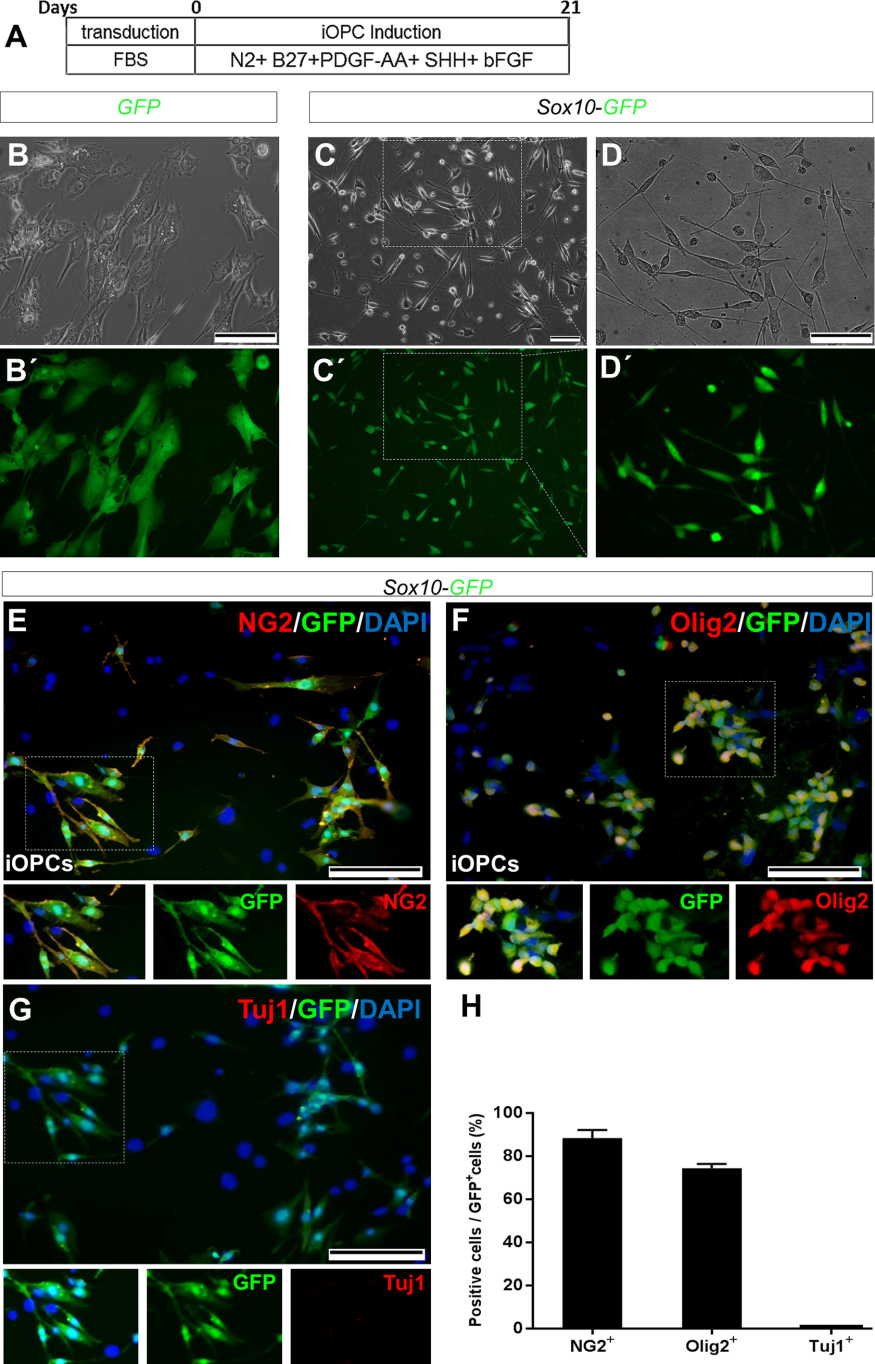
Changes in morphological marker proteins expression during generation of Sox10-induced oligodendrocyte progenitor-like cells (OPCs) from astrocytes and Characterization of induced OPCs. A) Schematic time diagram and the content of culture medium for in vitro generation of induced oligodendrocyte progenitor cells (iOPCs) from astrocytes via Sox10 expression. B, B´) Typical morphology of control astrocytes which were transduced with IRES-GFP vector. C, C´) Morphology of Sox10-transduced cells preserved in OPC induction medium for 21 (21 dpi). (D, D´) Magnified images of the white square in C, C´ which shows a bipolar morphology for induced cells. E-G) Immunofluorescence images of Sox10-IRES-GFP induced oligodendrocyte progenitor cells (iOPCs) stained with OPC markers NG2 (E) and Olig2 (F), and neuron marker Tuj1 (G) at 21 day post induction. In vitro induction of astrocytes to OPCs was repeated 3 times. Non-transduced cells in E and F (blue nuclei) did not express OPC markers. G) Quantification of NG2+, Olig2+ and Tuj1+ cells as percent of GFP+ cells. Cells were counterstained with DAPI. Scale bars: 50 μm.

To further support the process of in vivo reprograming of astrocytes by sox10, astrocytes were transduced in vitro by viral particles and subsequently cultured in OPC induction medium (Fig 6A). Approximately during 16–21 days after induction, we observed morphological changes in GFP-Sox10 vector transduced cells but not in GFP transduced astrocytes (Fig 6B–6D). Interestingly, in Sox10 treated cells, the large flat shape morphology shifted into bipolar spindle-like cells (Fig 6C and 6D) with proliferation capacity in response to PDGF-AA, bFGF and SHH. The induced cells showed expression of exogenous Sox10 as well as enhanced expression of OPC markers including Olig2, Myrf and endogenous Sox10 as assessed using RT-PCR. These cells did not express Krox20 a peripheral neural cell marker which showed the lack of Schwan cell differentiation. Astrocytes transduced with GFP vector did not express oligodendrocytes markers (Panel A in S4 Fig). The expressions of exogenous and endogenous Sox10 was measured using differentiating primers. Olig2 expression was increased during days 5–9 post induction (Panel B in S4 Fig). Compared to GFP vector transduced astrocytes, induced OPC-like cells showed decreased levels of Gfap and S100b expression (Panel C in S4 Fig).

Expressions of the OPC markers were confirmed at the protein level by ICF against NG2 and Olig2 (Fig 6E and 6F). Induced cells were negative for Tuj1 expression as a marker of neurons (Fig 6G). Quantification of positive cells showed that 88.33%±2.1 of GFP positive cells exposed to induction OPC medium were NG2+, 74.33%±1.2 were Olig2+ and no Tuj1+ cell was detected (Fig 6H).

## Discussion

Sox10 is related to the SRY gene family that regulates myelin gene expression in embryonic and adult oligodendrogenesis [36]. This factor binds to the enhancer of genes important for myelination, such as Myrf [37], Olig2 [27], and Mbp [38], and promotes oligodendrocyte differentiation.

In situ conversion of reactive astrocytes present in the glial scars to progenitor cells such as OPCs suggests a new attitude toward myelin repair by replacement of reduced oligodendrocytes. This strategy also enhances repair potential by reducing the inhibitory components of glial scars. The outcomes of the current study have shown that astrocytes could possibly be converted into oligodendrocyte lineage cells by ectopic expression of a single transcription factor, Sox10. This finding supported previous reports where Sox10 in accordance with two other transcription factors reprogrammed rodent fibroblasts into iOPCs [25, 26]. Here, we used one out of the three previously reported transcription factors. Astrocytes have been used as the starting cells because they share a common embryonic origin with oligodendrocytes and contain extensive similarities in their epigenomes. In this study, we choose a single gene. A report showed the effectiveness of Sox10 to convert satellite glia into oligodendrocyte-like cells in the peripheral nervous system [27]. Several studies have shown the plasticity of astrocytes to reprogram into iPSCs [39, 40], NSCs [11], neuroblasts [12], and mature neurons [10][14]. The reactive astrocytes within active demyelinated lesions are hypertrophic and proliferative, with some neural stem cell-like properties [6] that make them prone for reprogramming to progenitors. In addition, it has been reported that Sox10 can suppress astrocytic differentiation of glial progenitors via antagonistic relationship with NFIA as a key astrocyte fate determinant factor [28]. This may explain why Sox10 positive cells extracted from early post-natal brains mainly differentiate to oligodendrocyte [29]. Therefore NFIA inhibition by Sox10 may provide a possible mechanism for conversion of transduced astrocytes to oligodendrocyte lineage cells.

Here we have attempted to show the possible potency of Sox10 for in situ conversion of reactive astrocytes to OPCs. We observed reactivation of astrocytes in the brains of mice treated with cuprizone for 12 weeks. The viral particles were delivered to the corpora callosa of mice in order to target the astrocytes. At 3 weeks after the injection, astrocytes comprised 72.75% of GFP+ cells that received GFP control vector which imply for the astrocyte fate of the major group of transduced cells. Of course it should be mentioned that some labeled astrocytes or oligodendroglial cells observed in this experiment might be originated from progenitor cells transduced by GFP expressing viral vector at the time of viral particles injection. Most GFP+ cells that received Sox10 expressing vector expressed OPC markers Olig2 (73.75%), PdgfRα (54%), and NG2 (74%). Therefore a shift from astrocytes to OPCs by Sox10 was possibly occurred. At 8 weeks after the injection, some of the transduced cells expressed Mbp and Plp, as markers of mature oligodendrocytes. While the expression of ectopic Sox10 contributed to the conversion of transduced cells to OPCs, the role of the internal microenvironment of the demyelinated lesion should not be overlooked. Our previous experiments showed that *in vivo* ectopic expression of MicroRNA 302/376 in astrocytes converted them to neuroblasts [15], whereas application of the same microRNA into the brains of cuprizone fed mice caused transdifferentiation of astrocytes to oligodendrocyte lineage cells [17] which indicate the determining role of microenvironment.

The enhanced number of OPCs 3 weeks after injection of the viral particles might be due to the conversion of astrocytes to OPCs; but other possibilities including proliferation of transduced OPCs and death of transduced astrocytes, or possible transduction of neural progenitors and their subsequent differentiation to OPCs by Sox10 might be plausible. Therefore, in order to prove conversion of astrocytes to OPCs, we extracted and purified astrocytes from the cortexes of P3-P5 mice. After transduction with viral particles that expressed GFP or GFP/Sox10, we transplanted the cells to the demyelinated mice brains and traced the transplanted cells at 3 weeks after transplantation. Results showed that transplanted cells expressed oligodendrocyte lineage cell markers, but in low amounts the astrocyte marker. Because of the very local conversion of astrocytes to oligodendrocytes within the injection site it was not expected that the transdifferentiated cells improve the animal behavior in Y-maze, while myelin staining for transplanted cells was observed. To support the proof of concept for the conversion of astrocytes to an oligodendrocyte lineage by Sox10, we attempted to show the possible potency of Sox10 for in vitro conversion of cultured astrocytes to OPCs. Our findings demonstrated that ectopic expression of Sox10 in astrocytes led to the expression of endogenous Sox10, Myrf and Olig2 in vitro. Expression of these key transcription factors was accompanied with supporting morphological changes.

In this study we used a continuous active promoter to express Sox10, however the endogenous Sox10 and Olig2 locus became active in induced OPC-like cells as shown in S4 Fig; accordingly converted cells might be a stable lineage independent on the transgene. The controls, the GFP vector-transduced cells in the oligodendrocyte media, lacked the ability to transdifferentiate to OPC-like cells which showed a pivotal role for Sox10. Sox10 function highly depends on its expression level [41]. However, we were unable to manage the exact copy number of ectopic genes expressed in each cell. This might explain why a population of infected cells were not converted to oligodendrocyte lineage.

Ultimately, our proof-of-principle study may suggest the achievability of in vivo astrocyte conversion into oligodendrocyte-like cells using Sox10 gene delivery into the demyelinated lesions. Our data are promising for finding a new approach for myelin repair and astrocyte removal in patients with demyelinating insults.

## Supporting information

S1 Fig Schematic diagram of the expression vectors used in this study as well as the experimental procedure. A) The expression vector contained open reading frame sequences of mouse Sox10 and GFP under the control of a single promoter, SSFV. Control vector did not include Sox10 sequence. B) Schematic diagram of the experimental procedure for inducing demyelination, Y maze test for data presented in Fig 1D and 1E, virus injection, and tissue sampling.
(TIF)

S2 Fig The morphology and characterization of isolated astrocytes before sorting. A) immunocytofluorescence (ICF) showed that isolated astrocytes were positive for the glial fibrillary acidic protein (Gfap). We observed few Nestin (B), NG2 (C) and Olig2 (D) positive cells in cultured primary astrocytes. Scale bars: 100 μm.
(TIF)

S3 Fig Determining the purity of sorted astrocytes. After sorting the astrocytes using anti-CD44 antibody, we checked the purity of sorted astrocytes by immunocytofluorescence (ICF). ICF showed that purified cells were positive for glial fibrillary acidic protein (Gfap) (A) and negative for neural stem cell marker, Nestin (B), oligodendrocyte lineage markers Olig2 (C), Sox10 (D) and NG2 (E). Scale bars: 50 μm.
(TIF)

S4 Fig Induction of astrocytes to oligodendrocytes by exogenous Sox10 led to the expressions of endogenous OPC markers. A) Agarose gel electrophoresis analysis at day 21 after transduction confirmed the expression of endogenous Sox10, Olig2 and Myrf in cells transduced with Sox10-GFP vector, but not in astrocytes transduced with GFP vectors. B) Olig2 expression was increased in induced cells during days 5–9. C) Expression of Gfap and S100b as markers of astrocytes were reduced in induced OPC-like cells. Data in B and C was obtained using RT-qPCR. Primer specifications are provided in S2 Table.
(TIF)

S1 Table List of primary and secondary antibodies used in this study. (DOCX)

S2 Table Primer sets used for RT-qPCR analysis. (DOCX)
