# Supplementary figures and images for: In vivo conversion of astrocytes into oligodendrocyte lineage cells with transcription factor Sox10; Promise for myelin repair in multiple sclerosis
Source: PLoS One. 2018 Sep 13;13(9):e0203785. doi: 10.1371/journal.pone.0203785 (PMC6136770; doi:10.1371/journal.pone.0203785)

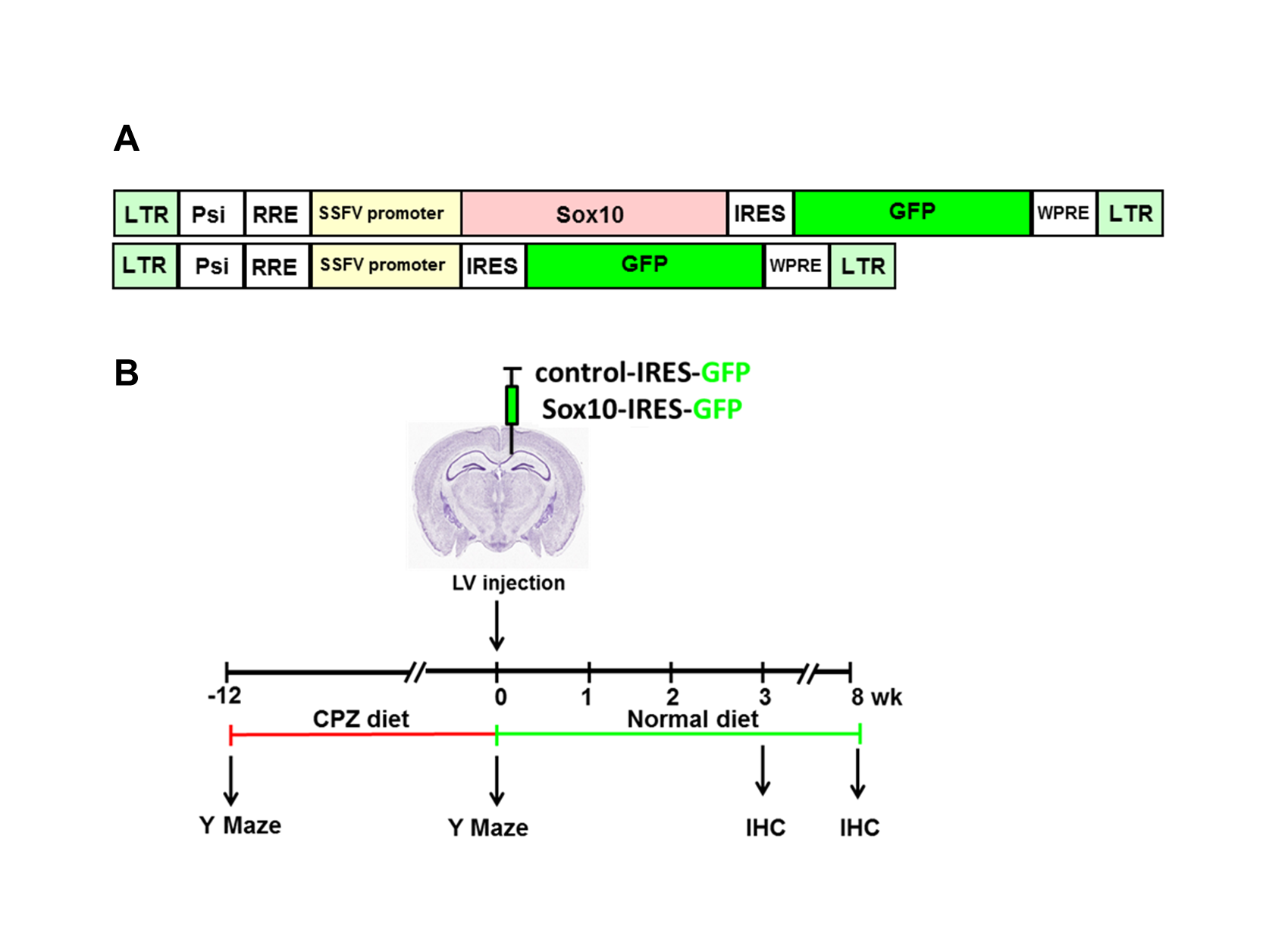

Supplement: S1 Fig — A) The expression vector contained open reading frame sequences of mouse Sox10 and GFP under the control of a single promoter, SSFV. Control vector did not include Sox10 sequence. B) Schematic diagram of the experimental procedure for inducing demyelination, Y maze test for data presented in Fig 1D and 1E, virus injection, and tissue sampling. (TIF) [file pone.0203785.s001.TIF]

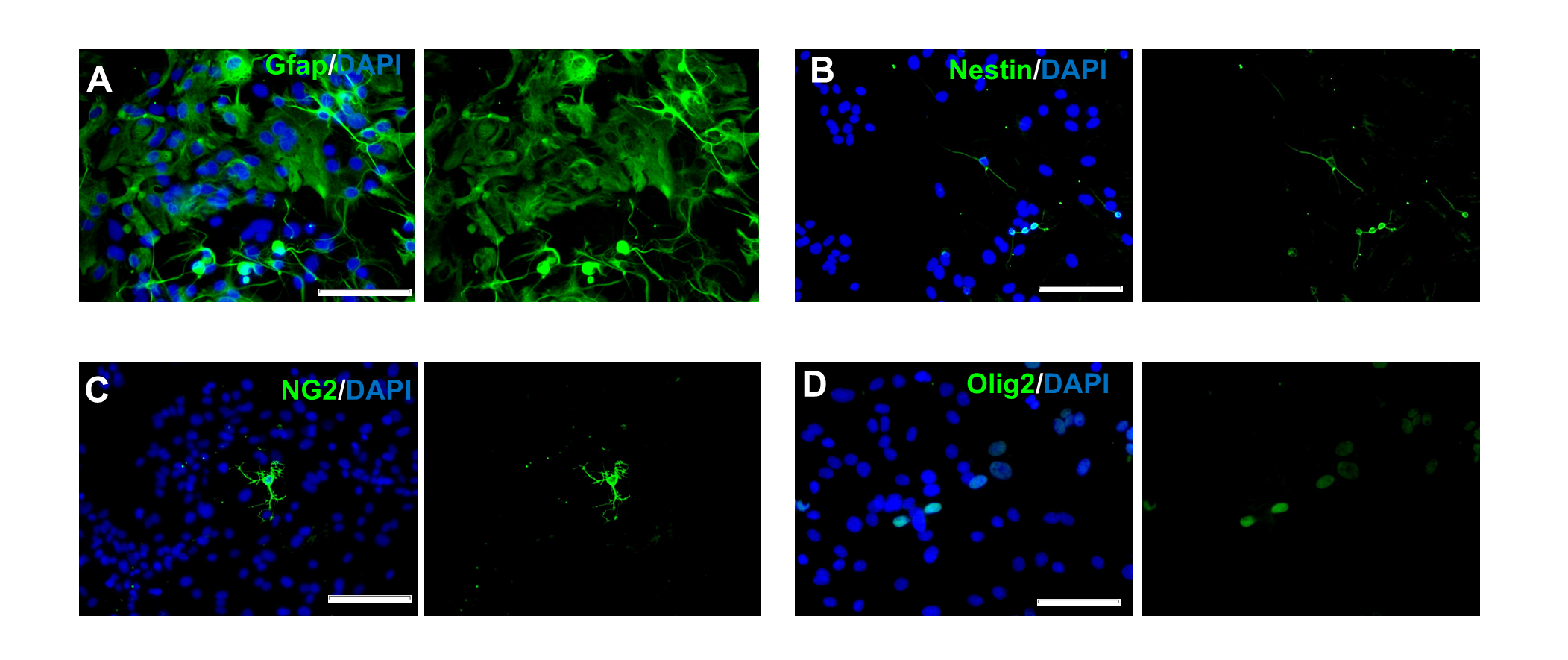

Supplement: S2 Fig — A) immunocytofluorescence (ICF) showed that isolated astrocytes were positive for the glial fibrillary acidic protein (Gfap). We observed few Nestin (B), NG2 (C) and Olig2 (D) positive cells in cultured primary astrocytes. Scale bars: 100 μm. (TIF) [file pone.0203785.s002.TIF]

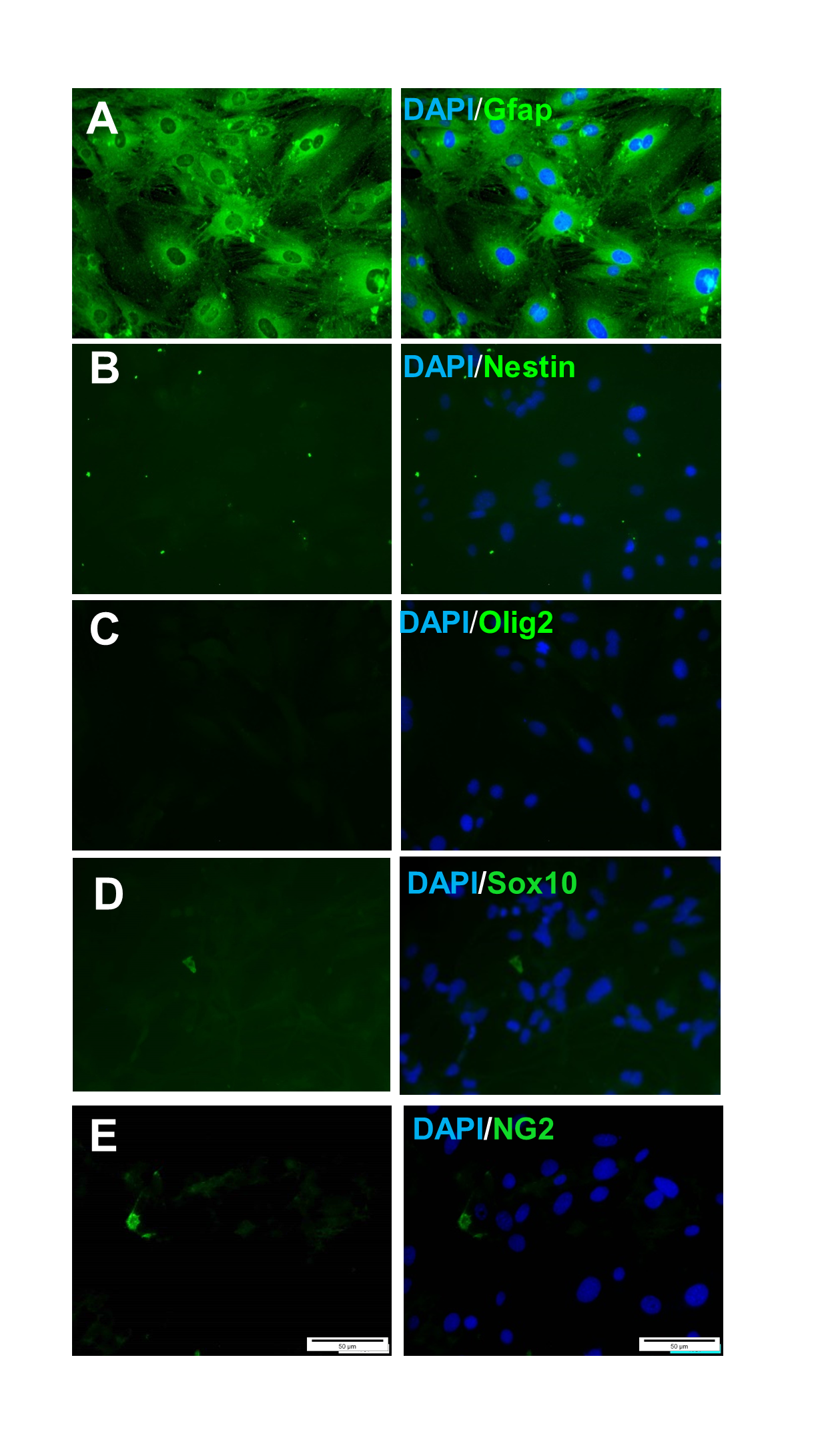

Supplement: S3 Fig — After sorting the astrocytes using anti-CD44 antibody, we checked the purity of sorted astrocytes by immunocytofluorescence (ICF). ICF showed that purified cells were positive for glial fibrillary acidic protein (Gfap) (A) and negative for neural stem cell marker, Nestin (B), oligodendrocyte lineage markers Olig2 (C), Sox10 (D) and NG2 (E). Scale bars: 50 μm. (TIF) [file pone.0203785.s003.TIF]

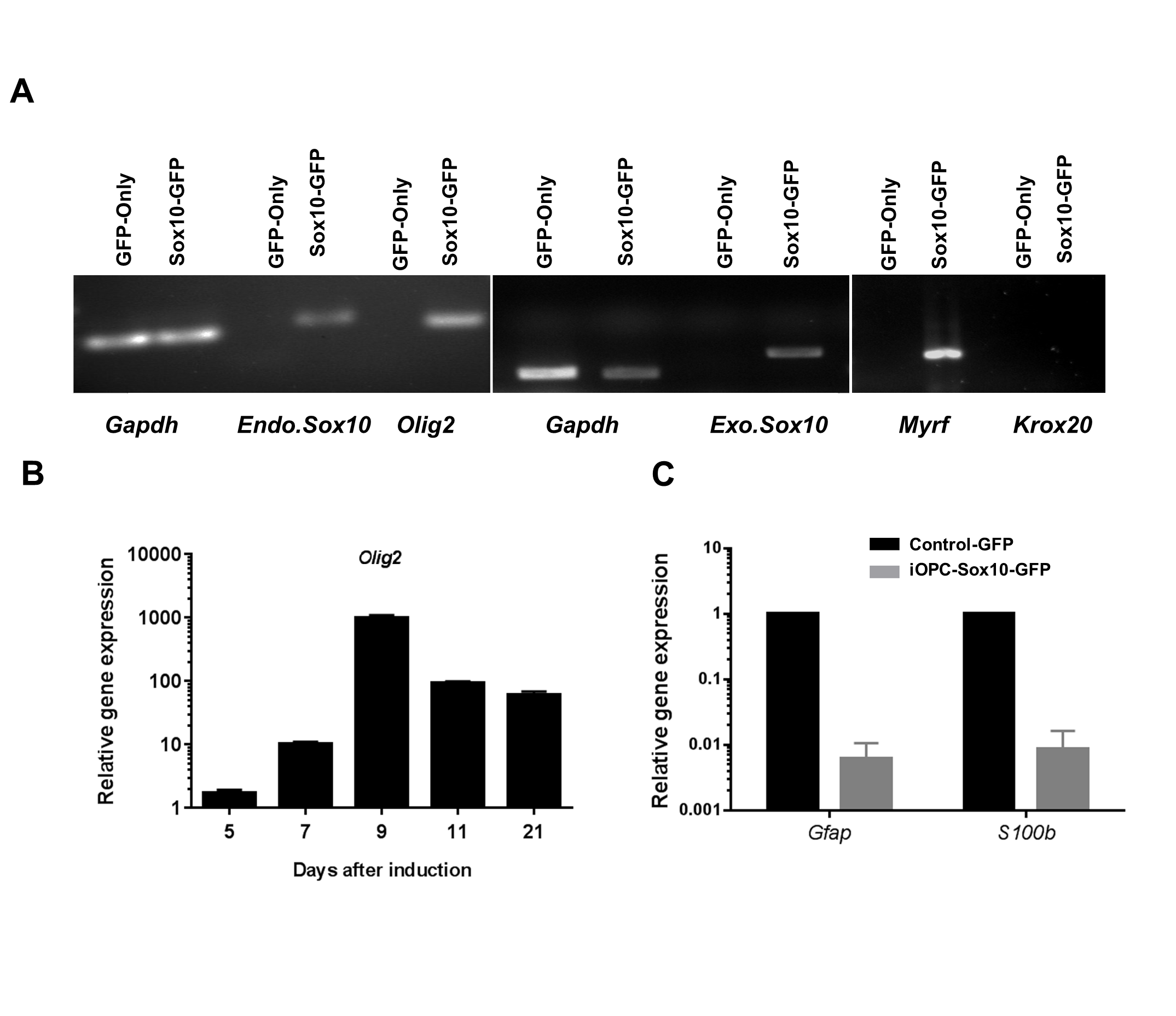

Supplement: S4 Fig — A) Agarose gel electrophoresis analysis at day 21 after transduction confirmed the expression of endogenous Sox10, Olig2 and Myrf in cells transduced with Sox10-GFP vector, but not in astrocytes transduced with GFP vectors. B) Olig2 expression was increased in induced cells during days 5–9. C) Expression of Gfap and S100b as markers of astrocytes were reduced in induced OPC-like cells. Data in B and C was obtained using RT-qPCR. Primer specifications are provided in S2 Table. (TIF) [file pone.0203785.s004.TIF]
