# Supplementary material for: In vivo conversion of astrocytes into oligodendrocyte lineage cells with transcription factor Sox10; Promise for myelin repair in multiple sclerosis
Source: PLoS One. 2018 Sep 13;13(9):e0203785. doi: 10.1371/journal.pone.0203785 (PMC6136770; doi:10.1371/journal.pone.0203785)
Supplement: S1 Table — (DOCX) [file pone.0203785.s005.docx]

**S1 Table. List of primary and secondary antibodies used in this study.**

| Antigen | Species | Catalog# | Condition | label |
| --- | --- | --- | --- | --- |
| CD44 | Rat | BD. 550538 | 1:100 |  |
| Anti-Rat IgG | Goat | MiltenyiBiotec: 120-000-290 | 1:500 | MicroBeads |
| Gfap | Mouse | Sigma: G3893 | 1:400 |  |
| Nestin | Rabbit | Sigma: SAB4200347 | 1:400 |  |
| Olig2 | Rabbit | Abcam: ab81093 | 1:200 |  |
| pdgfRα | Rabbit | Abcam: ab61219 | 1:100 |  |
| NG2 | Rabbit | Millipor: AB5320 | 1:100 |  |
| NeuN | Rabbit | Abcam: ab177487 | 1:500 |  |
| Mbp | Rat | Abcam: ab7349 | 1:200 |  |
| Plp | Rabbit | Abcam: ab28486 | 1:200 |  |
| Tuj1 | Rabbit | Abcam: ab6046 | 1:200 |  |
| Rabbit IgG | Donkey anti-rabbit | Invitrogen: A10040 | 1:1000 | Alexa Fluor® 546 |
| Mouse IgG | Goat anti-mouse | Invitrogen: A11004 | 1:1000 | Alexa Fluor® 568 |
| Rat IgG | Goat anti-rat | Invitrogen: A11081 | 1:1000 | Alexa Fluor® 546 |
