# Supplementary material for: In vivo conversion of astrocytes into oligodendrocyte lineage cells with transcription factor Sox10; Promise for myelin repair in multiple sclerosis
Source: PLoS One. 2018 Sep 13;13(9):e0203785. doi: 10.1371/journal.pone.0203785 (PMC6136770; doi:10.1371/journal.pone.0203785)
Supplement: S2 Table — (DOCX) [file pone.0203785.s006.docx]

**S2 Table: Primer sets used for RT-PCR.**

| Name of Genes | Forward | Revers |
| --- | --- | --- |
| *Gapdh* | 5' CAA CTC CCA CTC TTC CAC TT 3' | 5' GCAGCGAACTTTATTGATGGT A 3' |
| *Olig2* | 5'GAGCACCTCAAATCTAATTCAC3' | 5'AAAGATCATCGGGTTCTGG3' |
| *S100b* | *5' GATGTCTTCCACCAGTACTCC 3'* | *5'CTCATGTTCAAAGAACTCAT 3'* |
| *Gfap* | *5’-CTCCAAGATGAAACCAAC-3’* | *5’-GCAAACTTAGACCGATACC-3’* |
| *Endogenous Sox10* | *5' GGTGTTTGGTGGTGAGGATT3'* | *5' CTCGTCCTGAGGAAGTGGAA3'* |
| *Exogenous Sox10* | 5' AGCCCAGGTGAAGACAGAGA 3' | 5'CTCACATTGCCAAAAGACG3' |
| *Myrf* | 5' CCTGTGTCCGTGGTACTGTG 3' | 5'TCACACAGGCGGTAGAAGTG 3' |
| *Krox20* | 5' TTGACCAGATGAACGGAGTG 3' | 5'ACCAGGGTACTGTGGGTCAA 3' |
